# Supplementary material for: Exploring the chemical space of influenza neuraminidase inhibitors
Source: PeerJ. 2016 Apr 19;4:e1958. doi: 10.7717/peerj.1958 (PMC4841240; doi:10.7717/peerj.1958)
Supplement: Supplemental Information 1 [file peerj-04-1958-s001.pdf]

Supplementary Information

Exploring the chemical space of influenza neuraminidase inhibitors

Nuttapat Anuwongcharoen[1,2], Watshara Shoombuatong[1], Tanawut Tantimongcolwat[3], Virapong Prachayasittikul[2], and Chanin Nantasenamat*[1]

[1] *Center of Data Mining and Biomedical Informatics, Faculty of Medical Technology, Mahidol University, , Bangkok 10700, Thailand*

[2] *Department of Clinical Microbiology and Applied Technology, Faculty of Medical Technology, Mahidol University, , Bangkok 10700, Thailand*

[3] *Center for Research and Innovation, Faculty of Medical Technology, Mahidol University, , Bangkok 10700, Thailand*

*Corresponding author. E-mail: chanin.nan@mahidol.ac.th

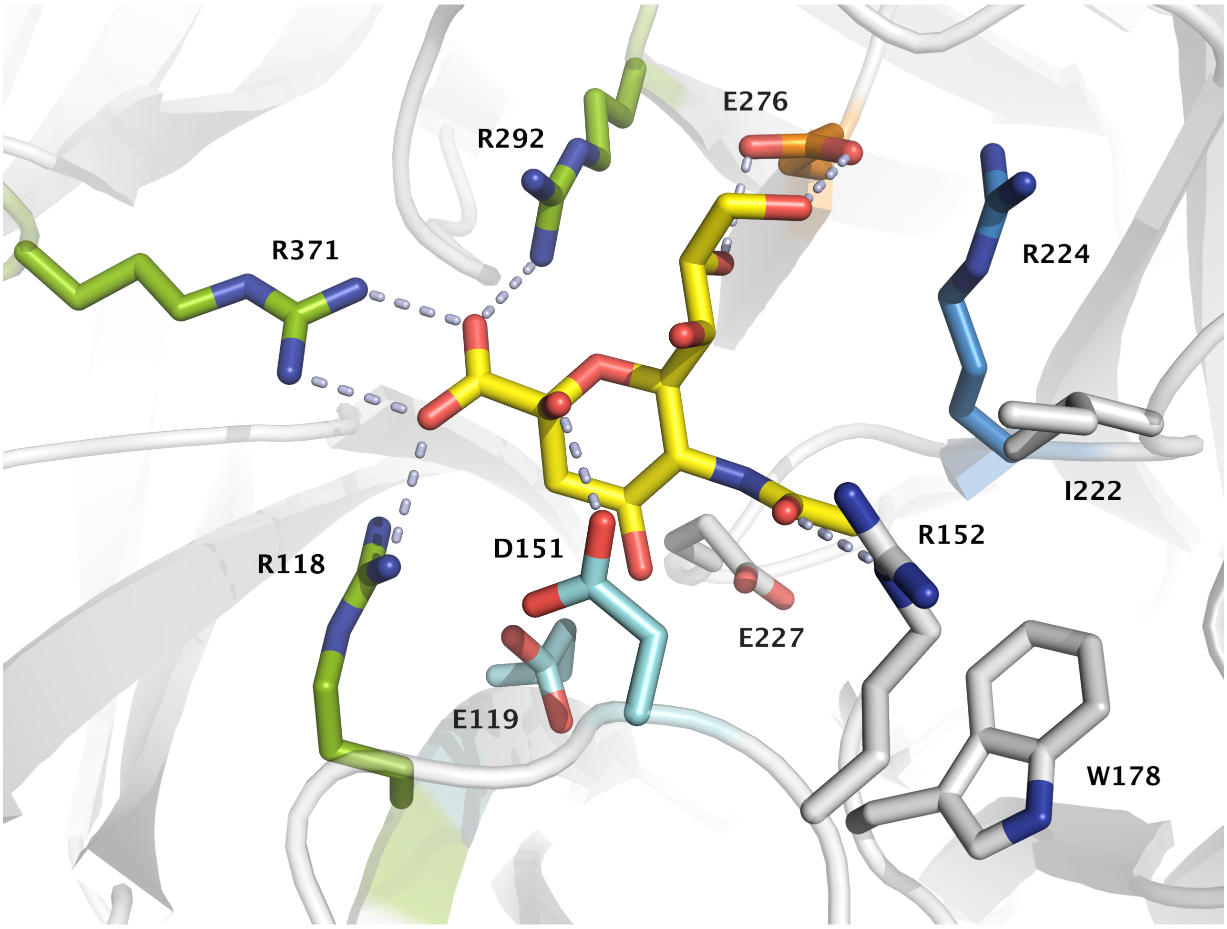

Supplementary Table 1: Binding mode of α-Neu5Ac with conserved amino acid residues inside the binding pocket of influenza A/N2 neuraminidase (PDB accession code 2BAT). Amino acid residues (N2 numbering) in subsites S1, S2, S3, S4 and S5 are colored green, cyan, white, blue and orange whereas the molecular interactions between ligand moieties and amino acid side chains are indicated by dashed lines.

Supplementary Table 1

Exploratory data analysis with the 6-term descriptive statistics of influenza A neuraminidase inhibitors.

**Statistics**	**MW**	**RBN**	**nCIC**	**nHDon**	**nHAcc**	**ALogP**	**TPSA**	Q_m	**Energy**	**Dipole moment**	**HOMO**	**LUMO**	**HOMO-LUMO**
**Active**													
Min	254.4	3.00	1.00	2.00	5.00	-3.669	90.65	0.139	-0.548	0.564	-0.373	-0.03	0.308
Q1	300.4	6.00	1.00	4.00	6.00	-0.619	101.65	0.151	-0.317	2.517	-0.36	-0.016	0.331
Median	332.4	7.00	1.00	4.00	8.00	0.076	121.96	0.161	-0.256	3.791	-0.352	-0.014	0.34
Mean	343.2	7.061	1.514	4.743	7.777	0.049	125.21	0.163	-0.274	4.101	-0.352	-0.006	0.346
Q3	372.3	8.00	2.00	6.00	9.00	0.595	148.61	0.169	-0.221	5.338	-0.345	-0.009	0.351
Max	665.9	15.00	5.00	9.00	11.00	5.614	200.72	0.272	0.00	9.977	-0.326	0.041	0.405
**Inactive**													
Min	145.2	1.00	0.00	1.00	1.00	-3.807	20.23	0.115	-0.681	0.208	-0.385	-0.066	0.272
Q1	265.3	3.00	1.00	3.00	6.00	-0.858	94.14	0.152	-0.301	2.82	-0.355	-0.033	0.301
Median	344.4	5.00	2.00	4.00	7.00	0.523	114.37	0.162	-0.237	4.176	-0.341	-0.026	0.316
Mean	328.4	5.248	2.109	4.321	7.204	0.853	120.03	0.179	-0.246	4.328	-0.343	-0.021	0.322
Q3	383.4	6.00	4.00	5.00	8.00	2.599	140.57	0.182	-0.172	5.459	-0.332	-0.015	0.339
Max	665.9	14.00	5.00	10.00	14.00	6.834	218.97	0.434	0.00	10.561	-0.311	0.044	0.403

Supplementary Table 2

Exploratory data analysis with the 6-term descriptive statistics of influenza B neuraminidase inhibitors.

**Statistics**	**MW**	**RBN**	**nCIC**	**nHDon**	**nHAcc**	**ALogP**	**TPSA**	Q_m	**Energy**	**Dipole moment**	**HOMO**	**LUMO**	**HOMO-LUMO**
**Active**													
Min	242.3	3.00	1.00	2.00	6.00	-3.669	95.66	0.139	-0.548	1.407	-0.368	-0.025	0.315
Q1	284.4	4.00	1.00	4.00	6.00	-0.722	95.66	0.148	-0.302	2.78	-0.358	-0.017	0.329
Median	300.4	6.00	1.00	4.00	6.00	0.18	101.65	0.157	-0.248	3.468	-0.353	-0.015	0.337
Mean	312.5	5.689	1.444	4.578	7.156	-0.128	114.59	0.158	-0.276	3.831	-0.351	-0.013	0.339
Q3	328.5	7.00	2.00	5.00	8.00	0.585	121.96	0.165	-0.207	4.811	-0.346	-0.01	0.345
Max	443.5	11.00	3.00	9.00	11.00	2.727	200.72	0.189	-0.135	8.19	-0.329	0.024	0.383
**Inactive**													
Min	194.2	2.00	1.00	2.00	5.00	-3.389	69.64	0.145	-0.679	0.564	-0.384	-0.033	0.297
Q1	309.8	5.25	1.00	4.00	8.00	-1.052	121.96	0.156	-0.301	2.94	-0.359	-0.019	0.326
Median	357.5	8.00	2.00	4.00	9.00	-0.205	135.6	0.166	-0.264	4.006	-0.35	-0.014	0.342
Mean	357.7	7.233	1.709	4.849	8.337	-0.017	134.17	0.168	-0.249	4.364	-0.35	-0.003	0.347
Q3	390.5	9.00	2.00	6.00	9.00	0.615	148.61	0.176	-0.195	5.782	-0.344	0.017	0.363
Max	665.9	14.00	5.00	10.00	10.00	5.614	192.7	0.272	0.00	9.791	-0.314	0.041	0.405
